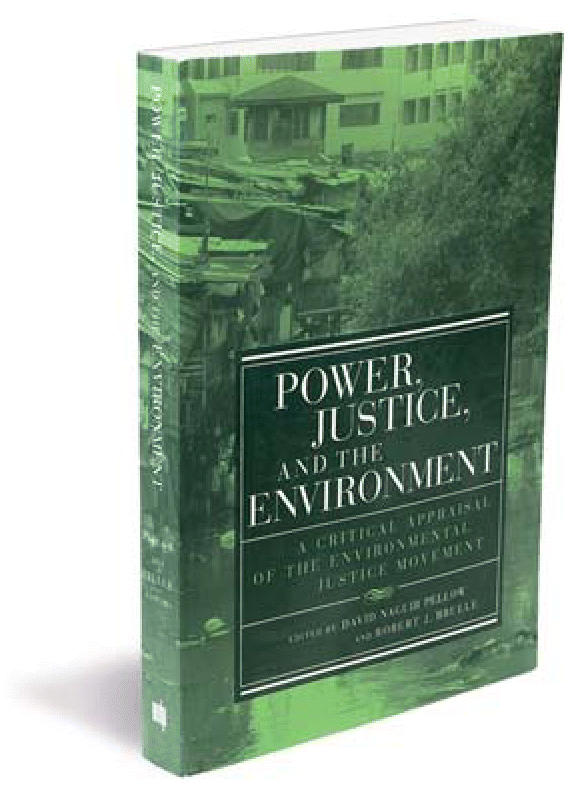# Power, Justice, and the Environment: A Critical Appraisal of the Environmental Justice Movement

**Published:** 2006-10

**Authors:** Peggy Shepard

**Affiliations:** Peggy Shepard is co-founder and executive director of West Harlem Environmental Action, founded in 1988 to improve environmental protection, health, and policy in communities of color. She is a member of the National Institute of Environmental Health Sciences Public Interest Liaison Advisory Group, and former chair, U.S. Environmental Protection Agency’s National Environmental Justice Advisory Council

Edited by David Naguib Pellow and Robert J. Brulle

Cambridge, MA:MIT Press, 2005. 339 pp. ISBN: 0-262-16233-4, $25

Over the past 18 years, hundreds of books have been published on the environmental justice movement (EJM); however, this is one of the first books to initiate a comprehensive dialogue that critiques strategies, tactics, and discursive frames; examine issues of organizational structure, governance, and resource base; assess goals and outcomes; and pose questions challenging academics and activists to consider where the movement has been and where it may go.

The EJM—which emerged in the late 1980s from struggles within communities of color and low-income communities that have been disproportionately affected by pollution—is characterized by editors Pellow, an activist–scholar who has published widely on EJ, and Brulle, an associate professor of environmental policy, as “a political response to the deterioration of the conditions of everyday life as society reinforces existing social inequities while exceeding the limits to growth. Thus the EJM laid a foundation for environmental and social justice politics in the twenty-first century.”

The heart and strength of these essays by academics, EJ practitioners, and advocates is the challenge to engage foundational concepts of the EJM that most serious observers and activists have been loath publicly to address. Yet 15 years since the historic first People of Color Environmental Leadership Summit, one reads this assessment and analysis and wonders why it has taken so long to begin this important inquiry.

The editors’ opening chapter succinctly summarizes literature on inequality, social justice movements, and environmental degradation, and presents provocative conclusions. Although Pellow and Brulle note that the EJM has affected the direction of environmental policy, research, and activism and that the EJM has had its “clearest victories” leading local community struggles, they question whether the EJM has achieved its goals and conclude that the “outlook is not positive.” They issue a challenge, echoed by the authors, to the EJM to “complement its well-honed acumen for opposition to unsustainable projects to a concrete vision and plan of action for construction and protection of sustainable communities”—a challenge that is being met by many EJ organizations.

The collection, targeted to scholars, theorists, practitioners, and activists, has three sections. In the first, “Environmental Quality and Justice: Progress or Retreat?” Bryant and Hockman compare the Civil Rights and EJ movements. Some chapters break the chain of synergy and some authors’ conclusions contradict others, but Benford’s “Half-Life of the Environmental Justice Frame: Innovation, Diffusion, and Stagnation” complements other authors in this book with his provocative argument that the “EJ frame suffers from stagnation as a result of its diffuse conceptualization, the many issues it seeks to address, the subordination of environmentalism to human justice, and its failure to embrace and articulate revolutionary solutions.” His argument needs more research and documentation to bolster his conclusion that the EJM’s “power lies in its capacity to disrupt the system rather than to reform it.”

Section 2, “New Strategies for Achieving Environmental Justice,” focuses on energy activism, sustainable and just food systems, case studies of collaborative problem solving, and research findings that the governance structure of EJ groups does not reflect its democracy frame. Brown offers lay-driven efforts to reframe social conceptions of health through the asthma/air quality paradigm, and Targ argues for a state-level comprehensive approach to EJ to address issues and approaches across agency jurisdictions. Pena urges that the EJM advance “exploration of autonomy”—self-governance of environmental management in local places—versus the struggle against “toxic racism and its rampant inequalities of place and power.”

The third section, “Environmental Justice and the Challenge of Globalization,” highlights South African perspectives, global activism initiatives, and the contrast between the goal of environmental sustainability, management of natural resources, and that of the EJM, human justice, wherein the natural environment is important only “inasmuch as it can be seen in terms of human justice.”

Brulle and Pellow conclude by advising EJ scholars to balance documentation of problems with a solution orientation, provide a stronger class analysis, and heed more of the links between EJ research and the literature on social movements. They warn EJ activists that forging a national agenda could deprioritize grassroots base building and that the divide between mainstream environmental groups and the EJM must be addressed more productively. They advise EJ groups to build a greater indigenous base of financial support and to appeal to a broader base of the population by addressing its race-based focus to be more inclusive of class, gender, working-class whites, and nation inequalities.

This book asks serious questions that have been raised by the EJM itself and continue to challenge its activists. Although this collection can be fascinating, there are some facile recommendations and observations made without sound basis. Nevertheless, this book is a vital addition to the literature, and it catalyzes a critical dialogue on our environmental future.

## Figures and Tables

**Figure:**